# Propofol post‐conditioning alleviates hepatic ischaemia reperfusion injury *via* BRG1‐mediated Nrf2/HO‐1 transcriptional activation in human and mice

**DOI:** 10.1111/jcmm.13279

**Published:** 2017-07-27

**Authors:** Mian Ge, Huixin Chen, Qianqian Zhu, Jun Cai, Chaojin Chen, Dongdong Yuan, Yi Jin, Weifeng Yao, Ziqing Hei

**Affiliations:** ^1^ Department of Anesthesiology The Third Affiliated Hospital Sun Yat‐sen University Guangzhou China; ^2^ Department of Pathology The Third Affiliated Hospital Sun Yat‐sen University Guangzhou China

**Keywords:** propofol, post‐conditioning, liver transplantation, ischaemia reperfusion injury, Brahma‐related gene 1, nuclear‐related factor 2, reactive oxygen species

## Abstract

To explore the effects of propofol post‐conditioning (PPC) on hepatic ischaemia/reperfusion injury (HIRI) and the potential mechanisms that might be involved in the interaction of Brahma‐related gene1(BRG1) and Nuclear‐related factor 2(Nrf2). Patients were randomized into PPC(*n* = 16) and non‐PPC(NPC)( *n* = 21) groups. Propofol(2 mg/kg) was infused within 10 min. of the onset of liver reperfusion during liver transplantation in the PPC group. Liver function tests, as well as Brg1, Nrf2, Heme oxygenase‐1(HO‐1) and NADPH:quinone oxidoreductase1(NQO1) expression levels were evaluated. CMV‐Brg1 mice were designed to investigate the role of Brg1 overexpression during HIRI. Brg1 and Nrf2 siRNA were used to examine the relationship between Brg1 and Nrf2/HO‐1 pathways in propofol‐mediated effects in a human hepatocyte(L02) hypoxia/reoxygenation(H/R) model. In patients, PPC attenuated both donor liver pathological and function injury, and reducing oxidative stress markers, compared to the NPC group, 24 hrs after surgery. PPC increased liver Brg1, Nrf2, HO‐1 and NQO1 expression. In mice, PPC reduced HIRI by decreasing liver oxidative stress and activating Nrf2/HO‐1 pathway, accompanied by up‐regulation of BRG1 expression. BRG1 overexpression activated Nrf2/HO‐1 transcription in CMV‐BRG1 mice during HIRI. *In vitro,* PPC significantly elevated expression of Nrf2, HO‐1 and NQO1, resulting in a reduction of cell DCFH‐DA and 8‐isoprostane levels and decreased lactate dehydrogenase levels, leading to an overall increase in cell viability. Moreover, the protective effects of propofol were partially abrogated in Nrf2‐knock‐down or BRG1‐knock‐down hepatocytes. Nrf2‐knock‐down drastically reduced protein expression of HO‐1 and NQO1, while Brg1‐knock‐down decreased HO‐1 expression. Propofol post‐conditioning alleviates HIRI through BRG1‐mediated Nrf2/HO‐1 transcriptional activation.

## Introduction

The primary cause of early allograft injury following orthotopic liver transplantation (OLT) or liver resection is HIRI, and is associated with allograft dysfunction and patient mortality 1. Formation of reactive oxygen species (ROS), as well as oxidative stress, is considered to be the main mechanisms contributing to HIRI and early allograft dysfunction 2, 3. Animal studies have demonstrated that antioxidants have the ability to ameliorate HIRI through increasing the expression of antioxidant enzymes 4, 5, 6, 7. Studies have suggested that enhancing antioxidant capacity may be a potential therapeutic approach to reducing the incidence of HIRI.

Indeed, repeated ischaemic conditioning and several pharmacological agents have been shown to enhance hepatic antioxidative abilities and alleviate HIRI 3, 8, 9. However, this approach is often challenging due to the inherent complexities associated with surgery. Furthermore, ischaemic pre‐conditioning in cirrhotic patients may be poorly tolerated. Therefore, post‐conditioning with drugs to protect transplanted allografts against HIRI during OLT or liver resection would be a more desirable approach.

To date, there are no reports examining the potential of preventing HIRI with pharmacological agents such as propofol. Some studies have demonstrated, in a post‐conditioning procedure, the protective effects of propofol, which is an intravenous anaesthetic that has antioxidant properties 10. Furthermore, propofol has been demonstrated to be protective against I/R injury in both the heart and brain 11, 12. In addition, propofol has been shown to possess a variety of pharmacological functions, including enhancing expression of antioxidant enzymes, such as heme oxygenase 1 (HO‐1) and NADPH:quinone oxidoreductase 1 (NQO1) 13, 14, 15, 16. However, the mechanism by which propofol up‐regulates antioxidant enzyme expression remains unclear. It has been previously demonstrated that the nuclear transcription factor, nuclear‐related factor2 (Nrf2), mediates antioxidant response by mediating various regulatory components 17. BRG1 is a central component of the SWI/SNF chromatin‐remodelling complex that features a bromodomain and helicase/ATPase activity. It has been demonstrated that Nrf2 induces HO‐1 gene expression by recruiting BRG1 to HO‐1 regulatory regions in SW480, SW13 and 293T cells 18. However, whether propofol up‐regulates antioxidant enzyme expression through transcriptional regulation during OLT remains unknown.

This prompted us to observe the effects of post‐conditioning with propofol on HIRI during human OLT, and to explore the mechanisms by which HO‐1 is up‐regulated using animal and cellular models.

## Materials and methods

### Clinical experiment design

This study was approved by the Research Ethics Board of the Third Affiliated Hospital, Sun Yat‐sen University, China and registered with the Chinese Clinical Trial Registry at www.chictr.org.cn on 19th April 2013 (*No. ChiCTR‐OCH‐12002255*). This prospective randomized controlled trial was conducted in the Third Affiliated Hospital of Sun Yat‐sen University from May 2013 to May 2014. Thirty‐seven patients of American Society of Anesthesiologists (ASA) II to III, aged 18–65 years, scheduled for OLT, were enrolled in this study. Exclusion criteria were as follows: (*i*) absence of informed consent; (*ii*) functional damage of other critical organ systems; (*iii*) receiving a non‐cadaveric transplantation; (*iv*) hepatic re‐transplantation; (*v*) non‐functional donor liver; (*vi*) combined liver‐kidney transplantation.

Six patients with hepatic hemangiomas were included as the control group (Con). Healthy liver tissue adjacent to hemangiomas was used for comparative analyses.

Patients were randomized to propofol post‐conditioning group (PPC, *n* = 16) and control group (NPC, *n* = 21). Randomization took place shortly before surgery. Patients, laboratory personnel, outcome adjudicators and data analysers were blind to allocation. General anaesthesia was performed on all patients. Midazolam (0.05 mg/kg), sufentanil (0.3 μg/kg), propofol (1.5 mg/kg), cisatracurium (0.2 mg/kg) were used for rapid induction of general anaesthesia. Endotracheal intubation was performed 5 min. after induction. Inhaled sevoflurane (0.8–1.5 minimum alveolar concentration), sufentanil (0.1 μg/kg/hrs) and intravenous cisatracurium (0.2 mg/kg/hrs) were used to maintain moderate analgesia and muscle relaxation during surgery. Propofol (2 mg/kg) (PPC group) or the same volume normal saline (NPC group) was injected by intravenous pump within 10 min. following onset of reperfusion. Liver transaminase (AST, ALT), total bilirubin (TBIL) and prothrombin time (PT) were measured preoperatively, at the end of the surgery, and 6, 24 hrs, 2, 3, 5, 7 and 14 days after surgery. Oxidative stress levels were measured preoperatively, 3 hrs after reperfusion, 1, 3, 5 and 7 days after surgery. Liver tissue samples taken 3 hrs after reperfusion were dehydrated and embedded in paraffin. Tissues stained with haematoxylin–eosin (H&E) were observed under a microscope and scored by a pathologist 19 who was blinded for the study context.

### Animals and hepatic ischaemia reperfusion model

C57BL/6 mice (20–28 g) were obtained from Shanghai SLAC Laboratory Animal Co. Ltd. Brahma‐related gene‐1 (BRG1, NM_011417.3) transgenic (CMV‐BRG1) mice (male, 6–8 weeks, 20–28 *g*) were obtained from Cyagen Biosciences Inc. pRP.ExSi‐CMV‐Brg1 vectors were constructed to obtain the cytomegalovirus (CMV)‐BRG1 mice, which were bred and screened by Southern blot analysis. Two lines (ExSi‐CMV‐Brg1‐24 and pRP.ExSi‐CMV‐Brg1‐45), that determined by RT‐PCR (Figure S1), among six established lines were used for further experiments. Genotypes of CMV‐BRG1 mice were identified by PCR analysis, and only commonly observed phenotypes were used in our current study. The primers used in our study included the following: BRG1 transgene PCR primer forward: 5′‐GCACCAAAATCAACGGGAC‐3′, reverse: 5′‐CTAGGACCCAGCATTGCAC‐3′; Internal control PCR primer forward: 5′‐ACTCCAAGGCCACTTATCACC‐3′, reverse: 5′‐ATTGTTACCAACTGGGACGACA‐3′.

Forty‐two C57BL/6 mice were randomly divided into two groups (*n* = 21 for each group): the non‐HIR group underwent laparotomy but not hepatic ischaemia reperfusion, and the HIR group was subjected to 70% HIR model as described by Ke B *et al*. 20. The blood supply of the median and left hepatic lobes was occluded for 60 min., and reperfusion was initiated by clamp removal. Following reperfusion, both groups were randomly divided into three further subgroups (*n* = 7, for each group): control group (Con), lipid post‐conditioning (Lipid) and propofol post‐conditioning (Pro). These subgroups corresponded to intraperitoneal injections immediately after reperfusion, of equal volumes of solvent, lipid emulsion, and 40 mg/kg propofol, respectively. Twelve CMV‐BRG1 mice were randomly divided into sham and HIR group. Mice were killed after 6 hrs of reperfusion. Histology analyses were performed as described before. All animal experiments were approved by the Animal Ethical and Welfare Committee of Sun Yat‐sen University (Guangzhou, China).

### Liver function assessment

Blood samples from patients and animals were centrifuged (1500 g, 15 min., 4°C), and serum aspartate aminotransferase (AST) and alanine aminotransferase (ALT) levels were determined by a 7180 Biochemical Analyzer (Hitachi, Japan).

### Oxidative stress evaluation

Human serum was collected to assay levels of hydrogen peroxide (H_2_O_2_) (Keygen Biotech. Co., Ltd., Nanjing, China), and malondialdehyde (MDA) (Nanjing Jiancheng Bioengineering Institute, Nanjing, China). Mouse liver tissues were prepared as 10% tissue homogenates and centrifuged. Levels of superoxide dismutase (SOD) (Keygen Biotech. Co., Ltd.) and 8‐isoprostane (Cayman Chemical Company, Ann Arbor, Michigan, USA) were determined according to the manufacturer's instruction.

### Immunofluorescence staining

Thawed sections of human liver tissue were washed three times with phosphate‐buffered saline (PBS) and fixed with PBS containing 0.3% Triton X‐100 and 5% bovine serum albumin for 1 hr, and then incubated with anti‐Nrf2(1:150) antibodies at 4°C overnight. Forty‐eight hours later, the slides were incubated with a fluorescently labelled secondary antibody (1:100) for 1 hr at 25°C after washing with PBS. The cover slips were washed again, mounted with mounting medium (Applygen, Biejing. No. 01210), and observed through a fluorescent microscope (Leica, DMLB2, Germany).

### Cell Culture and Hypoxia/Reoxygenation (H/R) model

Human hepatocytes L02 (ATCC) were maintained according to the manufacturer's recommendations. Cells were placed in Galaxy 48R hypoxia incubator (Eppendorf Company, Hamburg, Germany) with hypoxic gas (5% CO_2_, 94% N_2_ and 1% O_2_) at 37°C. After hypoxia, cells were transferred to a 5% CO_2_ incubator for reoxygenation. Propofol, solvent or lipid emulsion was added to the medium after reoxygenation.

### Cytotoxicity assay

Propofol cytotoxicity in L02 cells was evaluated using a CCK‐8 assay (Keygen Biotech. Co., Ltd.) and lactate dehydrogenase (LDH) cytotoxicity assay (Roche Diagnostics, Indianapolis, IN, USA).

### Plasmids and transfection

Recombinant adenovirus were generated by homologous recombination and amplified in L02 cells. The Brg1recombinant adenovirus was diluted with DMEM/F12 cell culture medium and added directly to the cells (MOI = 10) at 50% confluence. Small interfering RNAs (siRNA) specific for human BRG1 (SASI‐Hs01‐00019365, Sigma‐Aldrich, St. Louis, MO, USA) and human Nrf2 (siB11731135640, RiboBIO) were transfected into cells at 50% confluence according to the manufacturer's instruction for 48 hrs. Transient transfections were performed with Lipofectamine™ 2000 (Invitrogen, Carlsbad, CA, USA).

### Reactive Oxygen Species (ROS) Production Assay

ROS production was assessed using 6‐carboxy‐2′7′‐dichlorodihydrofluorescein diacetate (DCFH‐DA) (Sigma‐Aldrich, D6883). After incubation, a 100 μl suspension of hepatocytes was washed in PBS and stained with DCFDA at a final concentration of 10 μM. Hepatocytes were incubated at 37°C for 30 min. in the dark and washed two times in PBS. The ROS‐dependent fluorescence was measured by fluorescence microscope (Olympus IX71, Tokyo, Japan) at a magnification of ×200 using identical acquisition settings for each section.

### Protein Extraction and Western blotting

Whole cell lysates and nuclear proteins were extracted as described in our previous study 13. Western blot analyses were performed with following primary antibodies, including anti‐BRG1 (Abcam Company, Cambridge, UK, ab110641), anti‐Nrf2 (Abcam company, Cambridge, UK, ab89443), anti‐HO‐1 (Santa Cruz Biotechnology, Texas, USA, SC‐10789), anti‐NQO1 (Santa Cruz Biotechnology, Texas, USA, SC‐32793), anti‐Lamin B2 (Cell Signaling Technology, Massachusetts, USA, #13823) and anti‐β‐actin (Cell Signaling Technology, Massachusetts, USA, #3700) antibodies.

### Statistical analysis

All data were analysed using SPSS 12.0 software (SPSS, Chicago, IL, USA). Normally distribution data were expressed as mean ± standard deviation, and the abnormally distribution data were expressed as median. The differences among the groups were evaluated using one‐way analysis of variance (anova) followed by the Tukey's test. Differences were determined to be statistically significant at *P <* 0.05.

## Results

### Propofol post‐conditioning attenuated liver injury in donor liver

**Figure 1:**
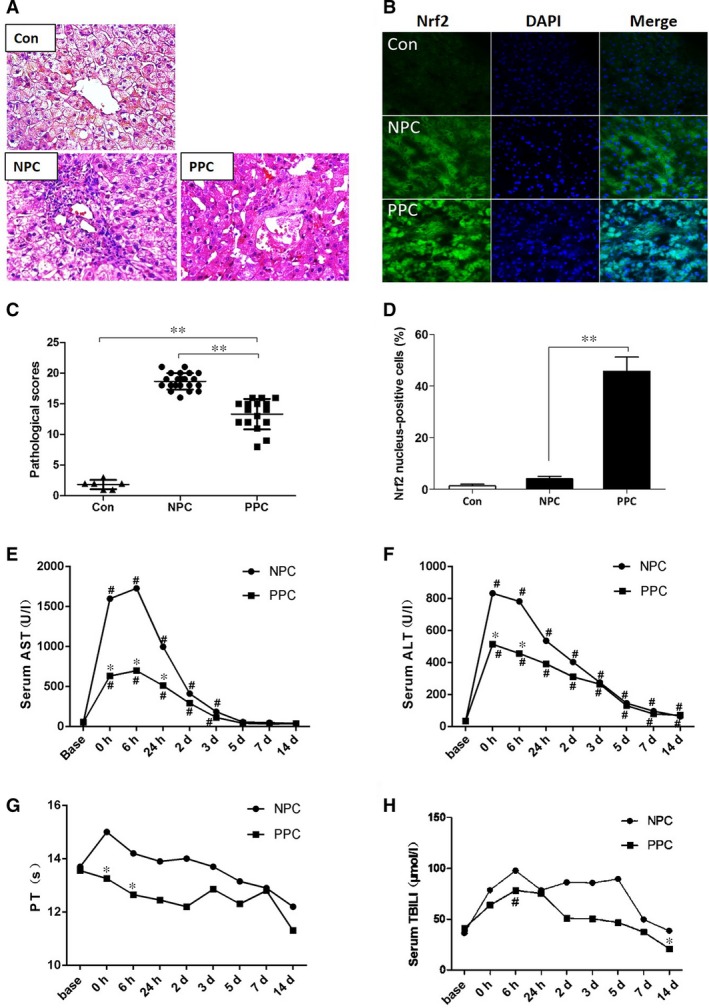
Propofol post‐conditioning attenuated liver injury in donor liver. (**A**) Liver tissues were stained with H&E, the pathology was observed under microscope (×400). (**B**) Immunofluorescence staining was performed with Nrf2 antibody, nuclei were stained with DAPI. (**C**) Suzike's injury score for liver damage degree. (**D**) Nucleus‐positive cells percentage of Nrf2. (**E**–**H**) Levels of aspartate aminotransferase (AST), alanine aminotransferase (ALT), prothrombin time (PT) and total bilirubin (TBIL) were measured preoperatively (base), at the end of the surgery (0 hrs), 6 hrs(6 hrs), 24 hrs(24 hrs), 2 days(2 days), 3 days(3 days), 5 days(5 days), 7 days(7 days) and 14 days(14 days) after surgery. The abnormally distribution data were expressed as median *P < 0.05 *versus* NPC group, **P < 0.01 versus NPC group, ^#^ *P <* 0.05 *versus* preoperatively conditions(base).

There were no statistical differences between PPC and NPC groups concerning preoperative and intra‐operative characteristics (Table 1). Allograft injury in the NPC group was of a severe level, with significant hepatocyte swelling, vacuolar degeneration, necrosis, sinusoidal congestion and massive lobular inflammatory cell infiltration, particularly in the portal areas (Fig. 1A). In contrast, pathological scores were less severe in the PPC group compared with NPC group (Fig. 1C). As shown in Figure 1B and D, immunofluorescence revealed weak nuclear staining for Nrf2 in the Con group. In the NPC group, OLT resulted in a small increase of Nrf2 translocation to the nucleus. Nrf2 expression in the nuclei further significantly increased after treatment with propofol. Similarly, ALT and AST activities were significant lower in PPC group than in NPC group within 24 hrs after surgery (*P <* 0.05 at the end of surgery, 6‐hr and 24‐hr post‐surgery for AST activities, and *P <* 0.05 at the end of the surgery and 6‐hr post‐surgery for ALT activities) (Fig. 1E and F). Prothrombin (PT), but not total bilirubin (TBIL) levels, was significantly lower in PPC group than in NPC group at the end of surgery, and 6‐hr post‐surgery (*P <* 0.05) (Fig. 1G and H). Both indexes were not significantly different between the two groups 24‐hr post‐surgery (*P >* 0.05).

**Table 1:** Pre‐operative and Intra‐operative condition

	NPC Group (*n* = 21)	PPC Group (*n* = 16)	*P*
Gender (M/F)	21/0	16/0	**‐**
Age (year)	48. 81 ± 8.30	48.12 ± 9.89	0.820
Height (cm)	168.10 ± 3.19	168.72 ± 4.75	0.636
Weight (kg)	60.28 ± 7.36	62.68 ± 9.53	0.393
BMI (kg/m^2^)	21.30 ± 2.16	21.96 ± 2.66	0.410
Principal diagnosis			
Liver cancer	12 (57.14%)	11 (68.75%)	0.515
Severe hepatitis and liver cirrhosis	5 (23.81%)	4 (25.00%)	1.000
Others	4 (19.05%)	1 (6.25%)	0.364
History of arterial hypertension	3 (14.28%)	3 (18.75%)	1.000
History of diabetes mellitus	4 (19.05%)	2 (12.50%)	0.680
History of smoking	2 (9.52%)	3 (18.75%)	0.634
History of alcohol	3 (14.28%)	1 (6.25%)	0.618
Child‐Pugh score	8.19 ± 2.69	7.31 ± 2.70	0.333
MELD score	30.03 ± 11.71	27.36 ± 9.01	0.456

As shown in Table 2, the percentage of patients whose AST levels returned to a normal range within 7 days after surgery in the PPC group was greater than in the NPC group (75.0% *versus* 33.3%, *P <* 0.05). ALT levels of nine (56.2%) PPC patients returned to normal range within 30 days after the surgery, compared to four (19.0%) NPC patients (*P <* 0.05). Eleven (68.7%) recipients regained normalized TBIL levels within 14 days after the surgery in the PPC group, *versus* only four (19.0%) in the NPC group (*P <* 0.05). Post‐operative PT normalization within 3 days was observed in 11 (68.7%) PPC patients compared with five patients (23.8%) in the NPC group (*P <* 0.05). These results indicated that propofol post‐conditioning accelerated the recovery of donor liver metabolism and synthetic function after the surgery.

**Table 2:** The speed of liver function return to normal

	NPC Group (*n* = 21)	PPC Group (*n* = 16)	*P*
Cases whose post‐operative AST return to normal within 7 days (%)	7 (33.3%)	12 (75.0%)	0.012*
Cases whose post‐operative ALT return to normal within 30 days (%)	4 (19.0%)	9 (56.2%)	0.019*
Cases whose post‐operative TBILI return to normal within 14 days (%)	4 (19.0%)	11 (68.7%)	0.002*
Cases whose post‐operative PT return to normal within 3 days (%)	5 (23.8%)	11 (68.7%)	0.006*

*indicate significant difference (*p*<0.05) between the groups.

### Propofol post‐conditioning reduced oxidative stress and up‐regulated the expression of BRG1, Nrf2, HO‐1 and NQO1 in donor livers

**Figure 2:**
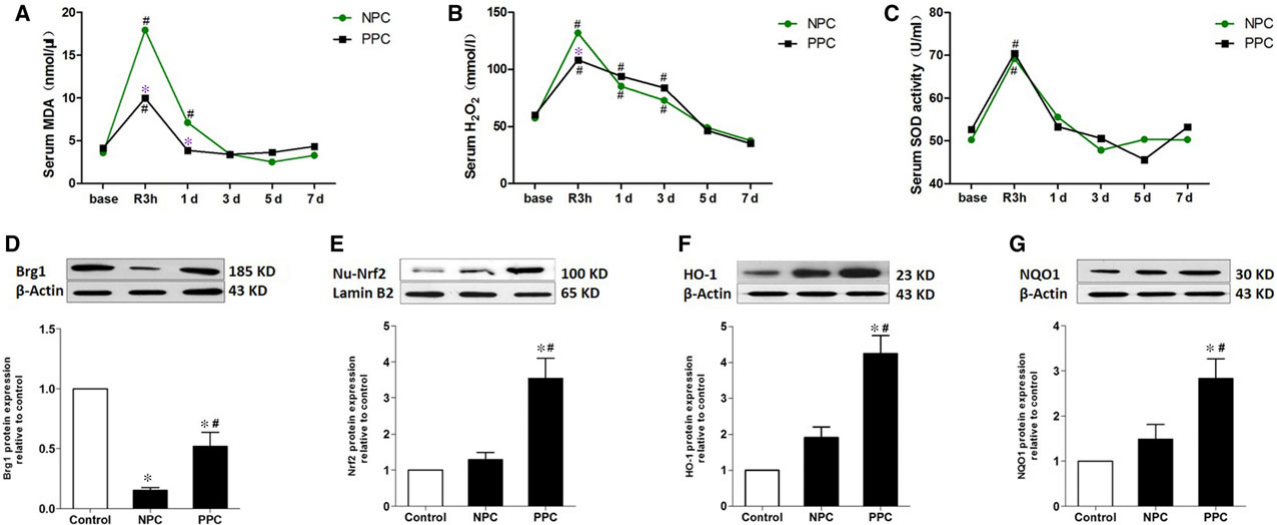
Propofol post‐conditioning reduced the level of oxidative stress and up‐regulated the expressions of BRG1, Nrf2, HO‐1 and NQO1 in donor liver. (**A**–**C**) The activities of malondialdehyde (MDA), hydrogen peroxide (H_2_O_2_) and superoxide dismutase (SOD) were measured preoperatively(base), 3 hrs after reperfusion(R3 h), 1 day(1 day), 3 days(3 days), 5 days(5 days) and 7 days(7 days) after surgery. *P < 0.05 *versus* NPC group, ^#^ *P <* 0.05 *versus* preoperatively conditions(base). (**D**–**G**) The expressions of Brg1, Nrf2, HO‐1 and NQO1 were assessed by Western blot. Normally distribution data were expressed as mean ± standard deviation, and the abnormally distribution data were expressed as median. *P < 0.05 *versus* control, ^#^ *P <* 0.05 *versus* NPC.

The expression levels of Nrf2 and its downstream target genes HO‐1 and NQO1 reflect antioxidative capacities. Nrf2 transcription previously been demonstrated to be influenced by BRG1 18. We examined whether the protective role of propofol post‐conditioning was related to Nrf2, its target genes or a regulatory factor. Compared with pre‐operative results, the levels of serum MDA, H_2_O_2_ and SOD activity were significantly increased 3 hrs after reperfusion (*P <* 0.05). An increase in H_2_O_2_ levels remained until 3‐day post‐operatively and then gradually declined to near pre‐operative levels, approximately five‐ to seven‐day post‐operatively (Fig. 2A‐C). Compared with the NPC group, PPC MDA levels were lower 3 hrs after reperfusion and 1‐day post‐operatively. H_2_O_2_ levels were lower at 3 hrs after reperfusion (*P <* 0.05), indicating that propofol post‐conditioning could inhibit oxidative stress in the early postoperative period following liver transplantation.

The results of Western blotting (Fig. 2D–G) showed that hepatocytes weakly expressed Nrf2 in healthy human liver tissue. Nrf2 expression in nucleus was increased following transplantation, but this increase was no statistical meaning. However, BRG1 expression decreased significantly 3 hrs after reperfusion compared with healthy liver tissue (*P <* 0.05). In comparison with the NPC group, the expressions of BRG1, Nrf2, HO‐1 and NQO1 in the PPC group were significantly higher (*P <* 0.05). These results demonstrate that propofol post‐conditioning promoted the expressions of BRG1, Nrf2, HO‐1 and NQO1 after reperfusion, which may be related to the protective effects of propofol.

### Propofol attenuated liver IR injury and up‐regulated the expression of BRG1, Nrf2, HO‐1 and NQO1 in mice

**Figure 3:**
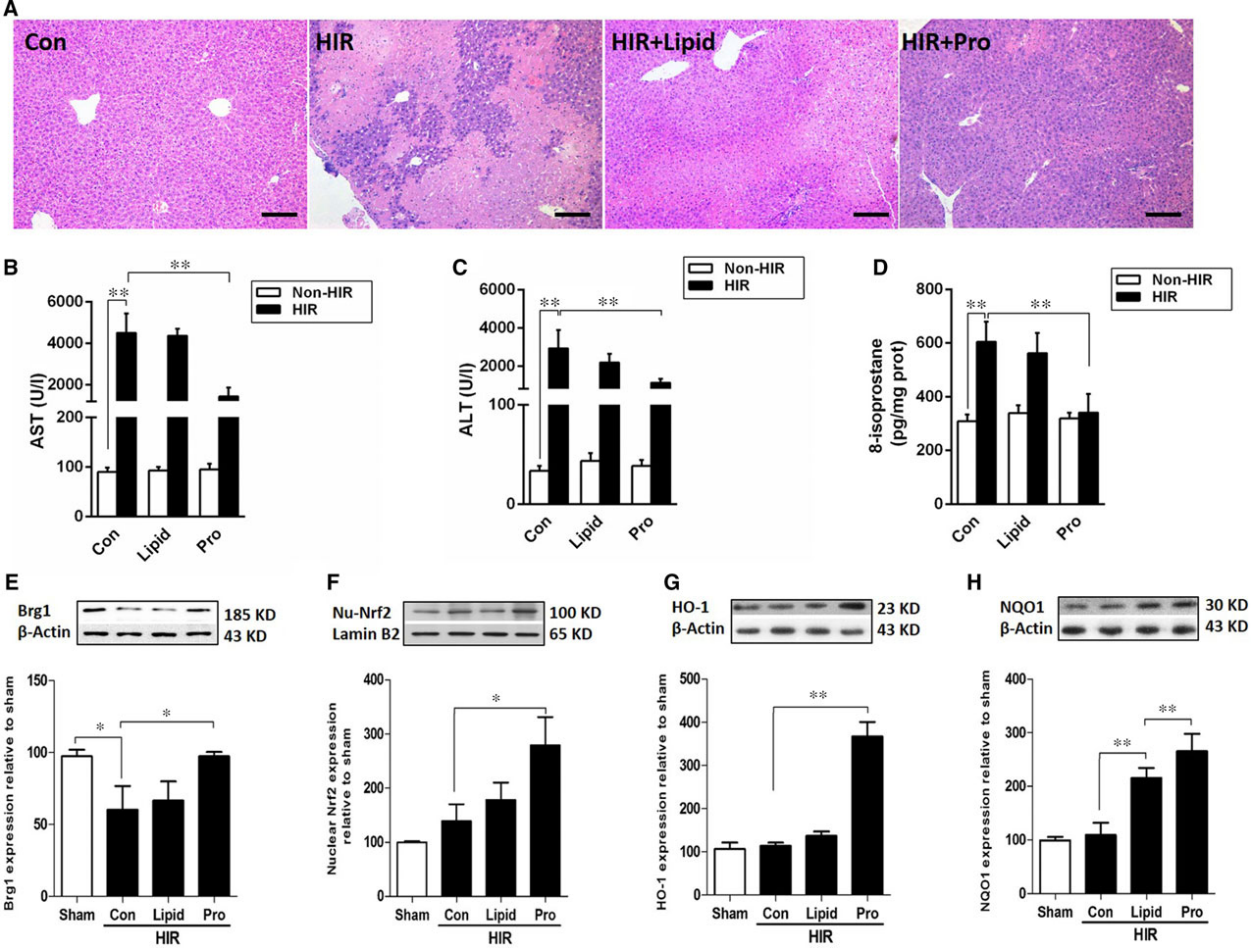
Propofol attenuated liver IR injury and up‐regulated the expression of BRG1, Nrf2, HO‐1 and NQO1 in mice. (**A**) Liver tissues of mice that were subjected to hepatic ischaemia reperfusion and treated with propofol were stained with H&E and observed under microscope (×100). (**B**–**D**) The levels of AST, ALT and 8‐isoprostane were measured. (**E**–**H**) The expressions of Brg1, Nrf2, HO‐1 and NQO1 were assessed by Western blot. *P < 0.05*,* **P < 0.01.

Having observed that propofol post‐conditioning exerted protective effects on liver transplantation, and promoted the up‐regulation of BRG1, Nrf2 and the Nrf2 target genes HO‐1 and NQO1, we further examined whether these findings were also apparent in animal models. Therefore, to examine this, mouse hepatic ischaemia reperfusion experiments were performed. Compared with mice in the HIR group, propofol post‐conditioning significantly attenuated hepatic pathological injury (Fig. 3A) and improved liver function, as indicated by a significant decrease in serum AST and ALT levels (*P <* 0.05, Fig. 3B and C). Moreover, the oxidative stress marker 8‐isoprostane was decreased (1‐fold, Fig. 3D) in the liver following propofol treatment, when compared to the HIR group. In addition, propofol significantly restored BRG1 protein expression (*P <* 0.05*,* Fig. 3E) and stimulated Nrf2 nuclear translocation, as shown by nuclear Nrf2 protein expression (Fig. 3F) and its downstream antioxidant enzymes HO‐1 and NQO1 (Fig. 3G and H), which were higher than the HIR group. Thus, these results showed that propofol prevented HIR injury due to antioxidative properties, which may be closely related to the Nrf2 pathway and BRG1 transcriptional regulation.

### BRG1 and Nrf2 gene knock‐down enhanced oxidative damage by H/R stimuli in L02 cells

**Figure 4:**
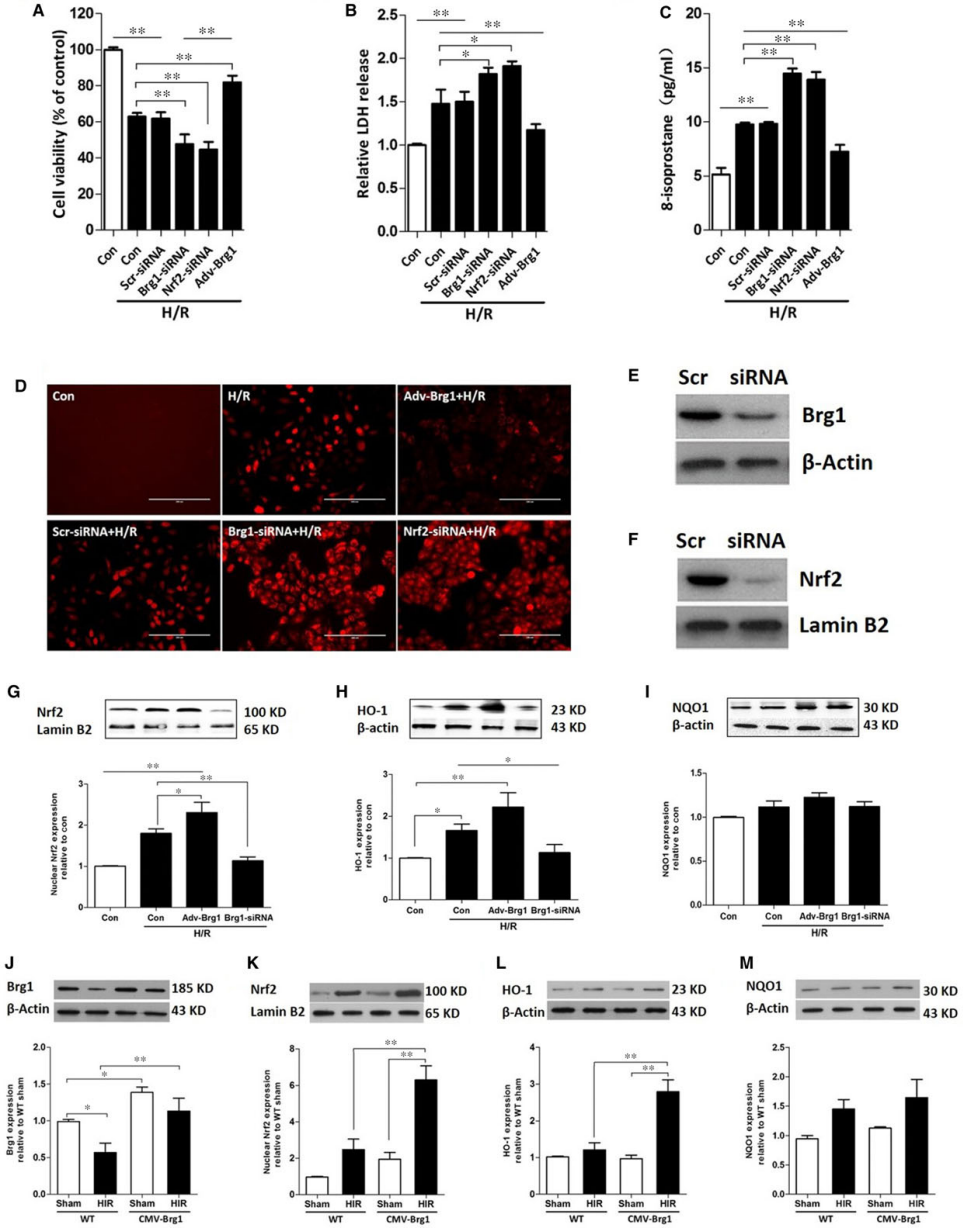
BRG1 and Nrf2 gene knock‐down enhanced oxidative damage by H/R stimuli in L02 cells. (**A**–**D**) L02 cells were transfected with siRNA Brg1 or siRNA Nrf2 or Adv‐Brg1 followed by Hypoxia/Reoxygenation (H/R) stimulation. Cell viability (**A**), LDH release (**B**) and the levels of 8‐isoprostane (**C**) were measured. (**D**) Dichlorofluorescin assay was used to test the production of intracellular ROS. Images were obtained by a fluorescence microscope, ×200. (**E**–**F**) The Brg1 and Nrf2 protein expressions were measured after transfected with Scr‐siRNA and Brg1/Nrf2 ‐siRNA. (**G**–**I**) L02 hepatocytes subjected to H/R stimulation, the expressions of Nrf2, HO‐1 and NQO1 were measured by Western blot after transfected with or without Adv‐Brg1 or Brg‐siRNA. (**J**–**M**) C57BL/6 mice and CMV‐Brg1 mice underwent sham operation or HIR, and the expressions of Brg1, Nrf2, HO‐1 and NQO1 were measured by Western blot. *P < 0.05, **P < 0.01.

To investigate the role of BRG1 in the Nrf2 pathway during liver ischaemia reperfusion, an *in vitro* study was carried out. As shown in Figure 4A and B, cell viability was reduced, and LDH levels were markedly increased in response to BRG1 and/or Nrf2 RNA interference. The excessive production of ROS was one of the most important factors mediating cellular hypoxia/reoxygenation injury. As shown in Figure 4C and D, 8‐isoprostane concentrations and intracellular ROS accumulation were significantly increased when subjected to H12R4 stimulation. A further increase was apparent in response to BRG1 and/or Nrf2 RNA interference, indicating that BRG1 and Nrf2 play an important role in ROS generation induced by hypoxia/reoxygenation. When BRG1 overexpressed though Adv‐Brg1 treatment in the H/R model, the protein expressions of Nrf2 and HO‐1 were increased, without significant changes in NQO1 expression (Fig. 4E–I). In parallel with these results, Nrf2 and HO‐1 expressions decreased after BRG1 expression was knocked down by siRNA in hepatocytes subjected to H/R (Fig. 4G–I). As showed in Figure 4J, the expression of Brg1 was higher in CMV‐Brg1 mice than that in WT mice in sham operation and HIR groups. When *in vivo* studies were performed (Fig. 4K–M), compared to WT HIR group and CMV‐Brg1 Sham group, Nrf2 and HO‐1 expressions were significantly increased in CMV‐Brg1 HIR mice; however, NQO1 expression did not exhibit significant changes.

### Propofol conferred protection in L02 cells subjected to H/R *via* Nrf2 and BRG1 activation

**Figure 5:**
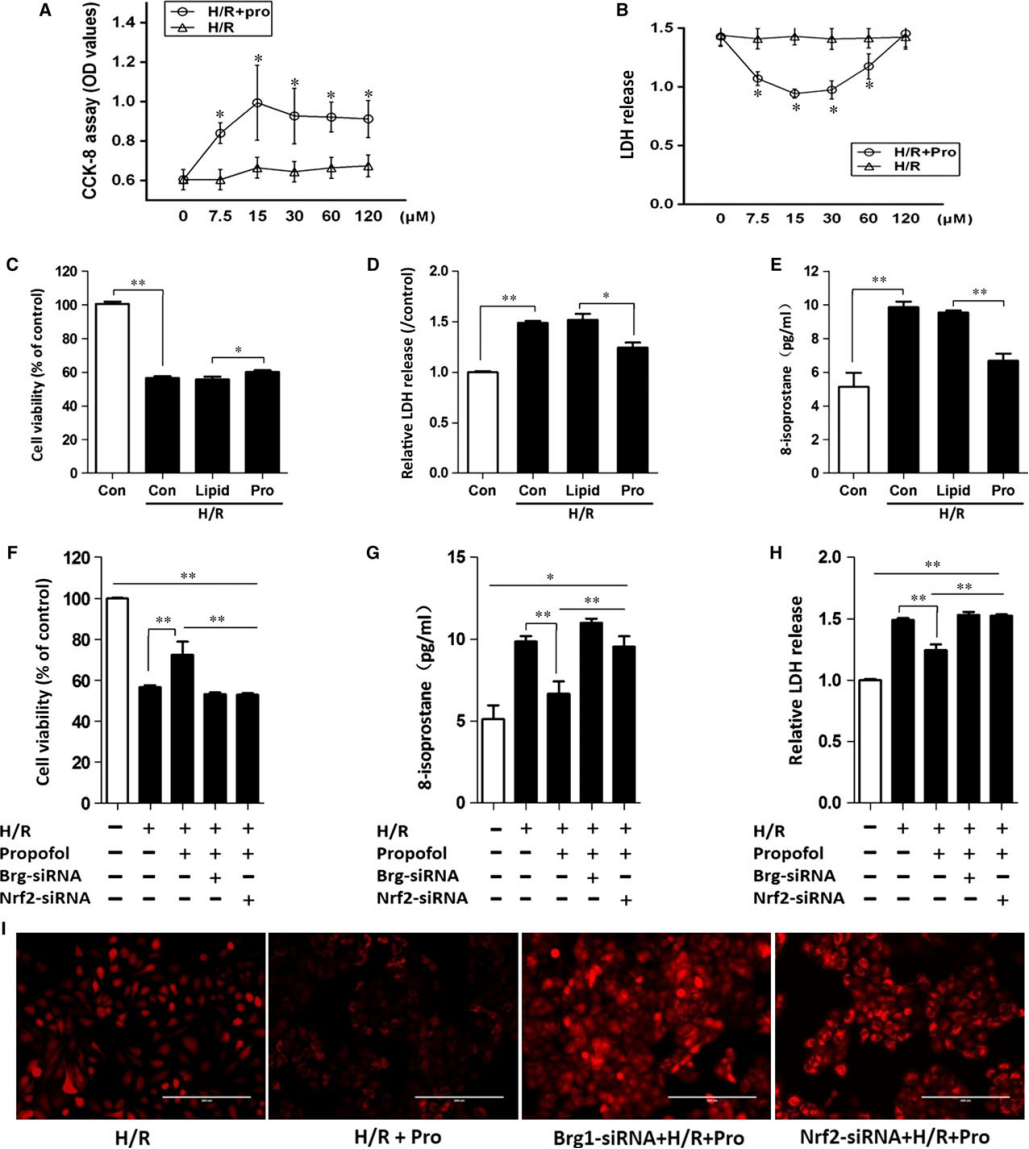
Propofol conferred protection in L02 cells subjected to H/R *via* Nrf2 and BRG1 activation. (**A**–**B**) L02 cells were treated with different concentrations of propofol medium after reoxygenation. Cell viability (**A**) and LDH release (**B**) were measured. (**C**–**E**) L02 cells were treated with lipid emulsion or 30 μM propofol in the H/R model. Cell viability (**C**), LDH release (**D**) and 8‐isoprostane (**E**) levels were assessed. (**F**–**I**) L02 cells were transfected with siRNA Brg1 or siRNA Nrf2 followed by Hypoxia/Reoxygenation (H/R) stimulation and propofol treatment. Cell viability (**F**), LDH release (**G**) and 8‐isoprostane (**H**) levels were determined. (**I**) Dichlorofluorescin assay was used to test the production of intracellular ROS when L02 cells were treated with or without propofol after transfected with Brg1/Nrf2‐siRNA in H/R model. Images were obtained by a fluorescence microscope, ×200. *P < 0.05, **P < 0.01.

**Figure 6:**
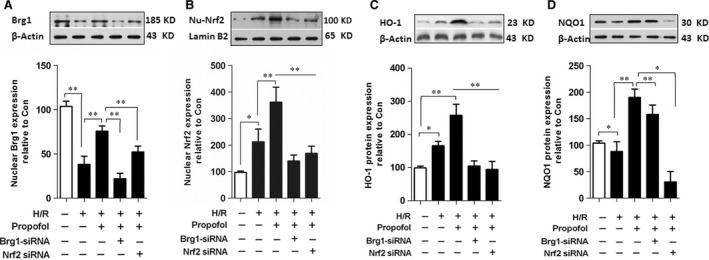
Propofol post‐condition elevated the expressions of BRG1, Nrf2 and its downstream genes. L02 cells were transfected with siRNA Brg1 or siRNA Nrf2 followed by Hypoxia/Reoxygenation (H/R) stimulation and propofol treatment. BRG1 (**A**), nuclear Nrf2 (**B**), HO‐1 (**C**) and NQO1 (**D**) expression was measured using Western blotting. *P < 0.05, **P < 0.01.

We then explored the underlying mechanism of propofol conferred hepatocyte protective effects against HIR injury, with a particular focus on BRG1 and Nrf2 changes. In line with findings from cell viability and LDH levels (Fig. 5A and B), 30 μM propofol exerted hepatocyte protection against H/R injury. Accordingly, this dose was chosen for our following study. Propofol treatment significantly elevated protein expression of Nrf2 and downstream HO‐1 and NQO1 (*P <* 0.05 *versus* Group H/R) (Fig. 6B–D). This was accompanied by an increase in BRG1 expression (Fig. 6A), resulting in a reduction of cell ROS intracellular accumulation (Fig. 5I) and 8‐isoprostane levels (Fig. 5E). Furthermore, cell viability also increased (Fig. 5C) and decreased LDH release (Fig. 5D). The protective effects of propofol were partially abrogated in Nrf2‐knock‐down and BRG1‐knock‐down hepatocytes using RNA interference. Nrf2‐knock‐down reduced protein expression of HO‐1 and NQO1, while BRG1‐knock‐down led to decreased HO‐1 expression, but did not affect NQO1 expression. These results indicated that propofol conferred protection in L02 cells subjected to H/R *via* both Nrf2 and BRG1 activation, which might provide synergistic effects on the production of downstream antioxidant enzymes.

## Discussion

In our present study, we confirmed that post‐conditioning with propofol alleviated allograft injury in OLT patients and HIRI in mice. In both clinical trial and animal experiments, propofol post‐conditioning reduced hepatic expression of oxidative stress, accompanied by the increased expression of Brg1, Nrf2, HO‐1 and NQO1. The cytological study confirmed that in response to oxidative stress, interactions between Nrf2/HO‐1 depend upon Brg1, while Nrf2/NQO1 was not.

Previous studies have indicated the protective effects of propofol post‐conditioning against I/R injury in both the heart and brain, while the effect of propofol post‐conditioning on HIRI remain unclear 11, 12. Our present data confirmed that propofol post‐conditioning protected the liver from HIRI in both clinical trial and animal experiments. Furthermore, in line with previous studies, our study also demonstrated that propofol enhanced HO‐1 and NQO1 expression [13, 14, 15, 16 ^]^. As a cytoprotective enzyme, HO‐1 shows anti‐inflammatory and anti‐oxidative effects. Oxidative stress and inflammation mediators induce HO‐1 gene expression 21, 22. Akin to HO‐1, NQO1 is a cytoprotective enzyme. HO‐1 and NQO1 are two main members of the Nrf2‐ARE system involved in the regulation of antioxidative responses [23 ^]^. The system exhibits antioxidant effects through up‐regulation of cytoprotective proteins including HO‐1 and NQO1, which are considered as the most powerful antioxidative enzymes, due to their relatively long half‐lives and low levels of degradation [24 ^]^. Thus, agents targeting the Nrf2‐ARE system could be considered as promising antioxidant and anti‐inflammatory modulators for conditions involving oxidative stress and inflammation, such as OLT and HIRI. Propofol has been shown to be protective in response to oxidative stress by promoting HO‐1 and NQQ1 expressions and by raising antioxidant capacity through an as yet, undefined mechanism 10, 12, 13, 14. Other studies have demonstrated that propofol pre‐conditioning increased Nrf2/GSH levels and attenuated lipopolysaccharide (LPS)‐induced production of ROS in human alveolar epithelial cells, while Nrf2 siRNA decreased the inhibition of propofol on ROS production 25. Therefore, propofol was most likely exerting its antioxidant capacity by acting on Nrf2 for HO‐1 and NQO1 up‐regulation. In line with this hypothesis, the results of our present study showed that propofol could induce the expressions of Nrf2, HO‐1 and NQQ1 in graft livers of patients and in mice suffering from IR.

However, the mechanism by which Nrf2 interacts with HO‐1 and NQQ1 remained unclear. A previous cytological study demonstrated that BRG1 was critical in Nrf2‐mediated inducible expression of HO‐1 in response to oxidative stress, while Nrf2‐mediated NQO1 induction was not BRG1 dependent 18. The interactions between Nrf2 and the two main Nrf2 regulated genes, *HO‐1* and *NQO1*, may be attributed to the different locations of the two genes 18. The interaction of Nrf2 with HO‐1 and NQQ1 was confirmed in our present study, which showed that propofol up‐regulated Nrf2 expression, leading to increased HO‐1 expression that was BRG1‐dependent, whereas inducible NQO1 expression was not. To the best of our knowledge, our study is the first to demonstrate that propofol post‐conditioning could alleviate IR through Nrf2‐HO‐1 in a BRG1‐dependent process.

Propofol is an intravenous anaesthetic, which has demonstrated protective effects against I/R injury in heart and brain through multiple mechanisms 11, 12, 26. Our present results confirmed that propofol post‐conditioning could protect the liver form I/R injury in association with the Nrf2‐HO‐1 system. However, in both clinical and pre‐clinical experiments, our results documented that increased expression of Nrf2, HO‐1 NQO1 and BRG1 by propofol did not fully protect the liver from I/R injury. It is probably related to the fact that BRG1 modulates the expression of a subset of activating and repressing genes involved in oxidative stress 18, 27. BRG1 is an important component of the mammalian chromatin‐remodelling complexes SWI/SNF, which utilizes ATP hydrolysis to alter nucleosome structure to promote or suppress gene expression 27, 28, 29. SWI/SNF complex interacts with different transcription factors to regulate the expression of multiple genes and modulates a wide range of cellular events 30, 31, 32. One study demonstrated that hypoxia up‐regulated the occupancies of Brg1 on promoters of cell adhesion molecules (CAMs) in a nuclear factor kappa B (NF‐ĸB)‐dependent manner 33. Thus, further investigations are needed to further dissect the molecular mechanisms involved in propofol post‐conditioning effects in response to oxidative stress. The results of our present study showed that propofol post‐conditioning represents a promising strategy to counteract HIRI, particularly in OLT and liver resection.

Several limitations to our study should be considered. First, the number of patients included in our clinical trial was small. Multicenter studies are, therefore, needed to confirm our results. Second, we did not explore the post‐conditioning effect of propofol in an OLT animal model, although the I/R model represented the pathological changes of OLT. However, the present conclusions are reinforced by the fact that our study which combined clinical, animal and cytological approaches, provided consistent results between these three approaches.

In summary, propofol post‐conditioning was able to ameliorate hepatic oxidative stress after hepatic ischaemia reperfusion by reducing tissue or intracellular ROS levels, and thus, confer protection in OLT patients. This favourable effect might be attributed to enhanced expression of Nrf2, HO‐1 and NQO1 by propofol that was confirmed in both clinical and experimental strategies. Furthermore, HO‐1 inducible expression of Nrf2 was BRG1 dependent, while BRG1 was dispensable for induction of another Nrf2 target gene, NQO1.

## Conflicts of interest

The authors have no conflicts of interests, financial or otherwise, related to the publication of this study or its findings.

## Supporting information


**Figure S1** Brg1 transgenic mice identification.
